# Abdominoscrotal Hydrocele With Intra-abdominal Undescended Testis in an Elderly: A Case Report

**DOI:** 10.7759/cureus.19520

**Published:** 2021-11-13

**Authors:** Fouad Hajji, Mohamed Amine Azami, Abderrazak Benazzouz, Nabil Hammoune, Omar Ghoundale

**Affiliations:** 1 Department of Urology, Caddi Ayyad University of Marrakech, Ibn Sina Military Hospital, Marrakesh, MAR; 2 Department of Pathology, Caddi Ayyad University of Marrakech, Ibn Sina Military Hospital, Marrakesh, MAR; 3 Department of Radiology, Caddi Ayyad University of Marrakech, Ibn Sina Military Hospital, Marrakesh, MAR

**Keywords:** hernia, aged adult, ultrasonography, cryptorchidism, undescended testis, abdominoscrotal hydrocele

## Abstract

Abdominoscrotal hydrocele (ASH) is an uncommon congenital anomaly in which a scrotal hydrocele extends to the abdomen through the inguinal canal in an hourglass fashion. Coexisting undescended testes (UDT) have mainly been reported in pediatric populations and are mostly located along the inguinal canal. We present a 66-year-old male with a history of neglected left cryptorchidism, who presented with a progressive ipsilateral inguino-scrotal swelling suggesting indirect inguinal hernia. On physical examination, inguino-scrotal hydrocele was suspected. Abdomen and pelvis computed tomography scan and magnetic resonance imaging revealed an abdominoscrotal cyst with a pathognomonic dumbbell appearance of an ASH, as well as an intra-abdominal testicle that proved to be intracystic, atrophic, and hypovascular. The patient underwent successful radical en-bloc excision of the ASH and testis via an extended inguinal approach. To our knowledge, this is the first case with this constellation of urogenital abnormalities to be reported in an aged man. What makes this case further unique and interesting is the unusual ASH’s relationship with the patient’s cryptorchid testicle and peritoneal sac.

## Introduction

Abdominoscrotal hydrocele (ASH), also known as hydrocele en bi-sac, is an uncommon variant of hydrocele of the tunica vaginalis testis, in which two cystic compartments (inguinoscrotal and abdominal) intercommunicate by an isthmus through the inguinal canal in an hourglass fashion [1,2]. Since the first ASH was described in 1834, there is a continued pattern of increase in pediatric ASH cases [3-5]; of all the 579 cases recorded through June 2018, only 208 cases have been documented in adults [5]. How this entity can come about remains unclear; displacement as per Laplace’s law, diverticulum theory, one-way valve effect, high infantile hydrocele, and imbalance between production and/or resorption of fluid have all been proposed as explanations [1-4]. The most widely accepted hypothesis is that increased pressure in the scrotal compartment may lead to its upward extension along the inguinal canal to form an intra-abdominal herniated sac that may lie retro or properitoneally. Associated conditions include contralateral hydrocele or inguinal hernia, contralateral or ipsilateral undescended testis (UDT), and have mainly been reported in pediatric patients [2,3]. This report describes an unusual case of ASH, which occurred in an aged man with a history of neglected ipsilateral UDT.

## Case presentation

A 66-year-old man with a 13-year history of uncontrolled type 2 diabetes mellitus was referred by his general practitioner to our outpatient clinic because of a clinical finding of reducible, non-complicated left-sided inguinoscrotal hernia.

The patient had noticed a progressive, painless swelling in the left groin area for the past three months, followed by a gradual swelling of the left hemiscrotum. He denied any history of change in bowel habits or voiding symptoms. He had six children and stated that its left hemiscrotum was always empty ever since he could remember.

**Figure 1:**
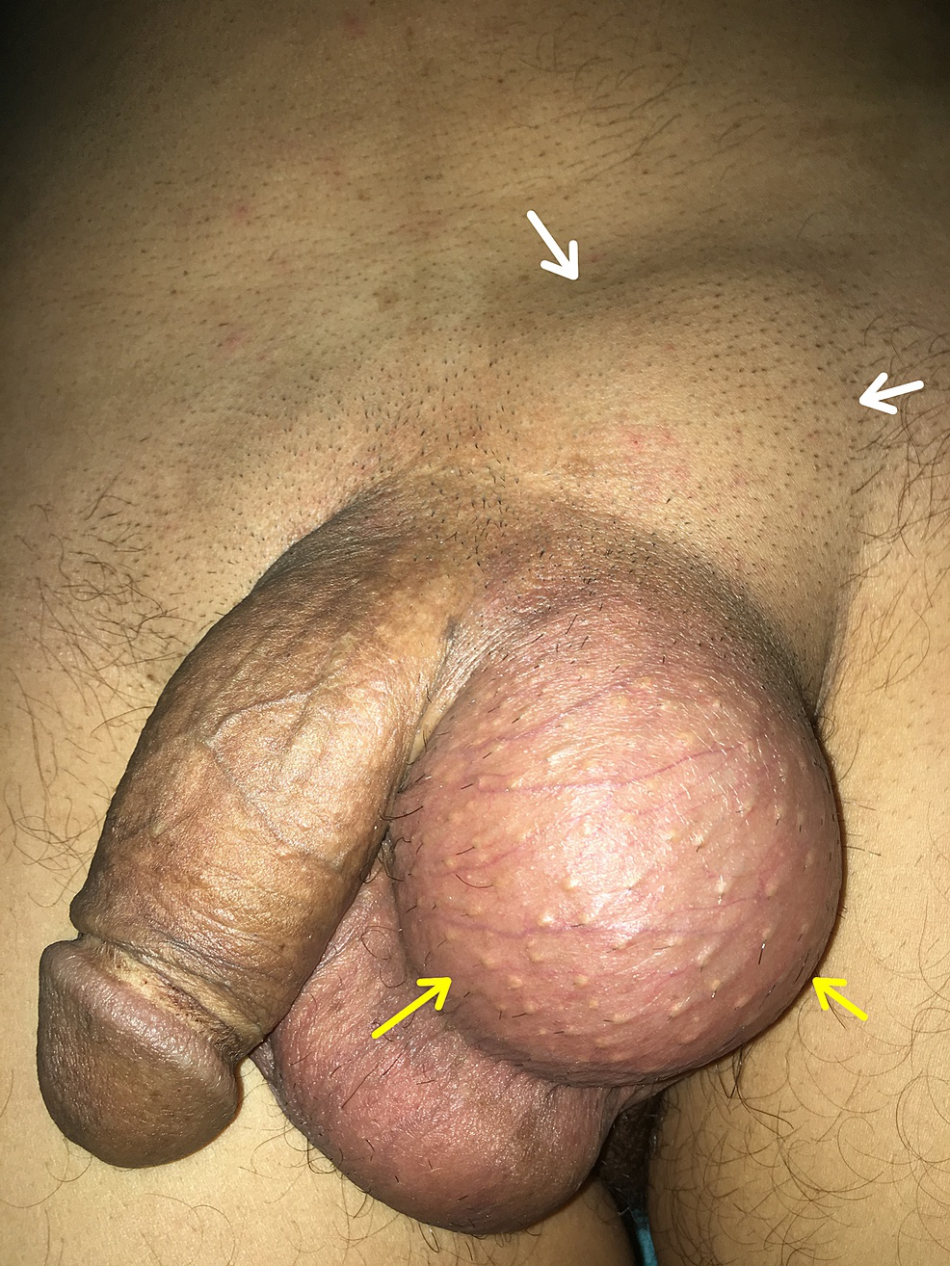
Photograph image showing scrotal (yellow arrows) and inguinal (white arrows) swellings with deviation of the penis on the opposite side.

His physical examination revealed a transilluminated, non-tender left scrotal swelling. There was a palpable, soft, and non-tender ill-circumscribed mass occupying the left inguinal region, with no palpable intraabdominal masses (Figure 1). The cross-fluctuation test was slightly positive with the scrotal swelling on bimanual examination, suggestive of inguinoscrotal hydrocele. The scrotal swelling was reducible with compression, making only the inguinal one expand, but the scrotum increased again once the pressure was released. Given the patient’s medical history, the testis tumor could not be ruled out. The right hemiscrotum was normal with a normally-positioned testis but without any clinical evidence of a hernia. The other system examinations were unremarkable.

Blood testing showed elevated levels of fasting plasma glucose (180mg/dL), glycated hemoglobin HbA1c (86.9 mmol/mol), blood urea nitrogen (15mmol/L), and creatinine (138 µmol/L) with reduced creatinine clearance (44.7ml/min). Testicular tumor markers (alpha-fetoprotein, beta-human chorionic gonadotropin, and lactate dehydrogenase) levels were within normal limits.

**Figure 2:**
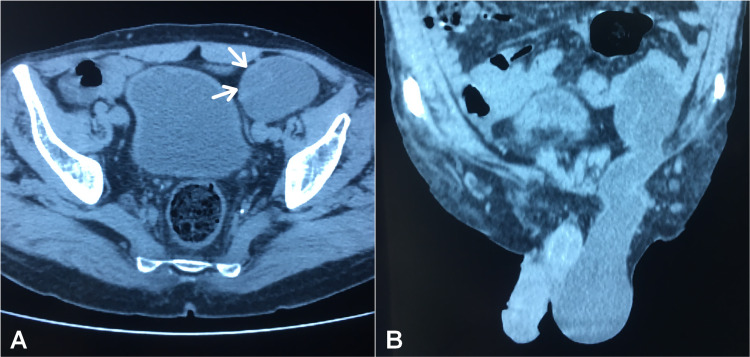
Abdominal and pelvic NCCT image showing an ASH and UDT. (A) Axial NCCT image showing a testis within the intra-abdominal sac (white arrows). (B) Coronal NCCT image showing hourglass appearance of ASH.

Non-contrast computed tomography (NCCT) of the abdomen and pelvis revealed a thin-walled, well-circumscribed cystic lesion of 11x53x48 mm, expanding from the left iliac fossa through the canal inguinal to the ipsilateral hemiscrotum in a dumbbell shape fashion, consistent with the diagnosis of ASH (Figure 2). The abdominal sac contained what was taught to be an intra-abdominal testicle. There were no ascites, lymphadenopathy, nor metastases, and the ipsilateral upper urinary tract was normal.

**Figure 3:**
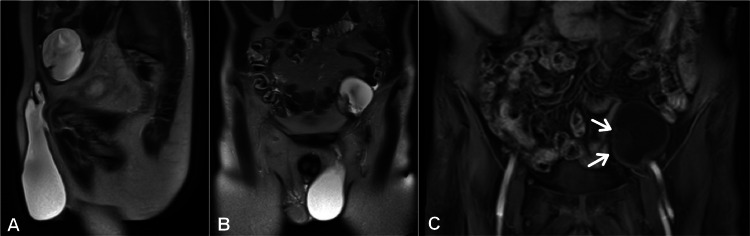
Abdominal and pelvic MRI image showing an ASH and UDT. (A) Sagittal and (B) Coronal T2-weighted MRI images showing dumbbell-shaped appearance of ASH. (C) Coronal contrast-enhanced T1-weighted MRI image showing an intracystic, atrophic and hypovascular testis (white arrows).

In order to investigate further, abdomen and pelvis magnetic resonance imaging (MRI) demonstrated an abdominoscrotal cyst with a hypovascular testis of 25x13x18 mm located within the abdominal sac (Figure 3). The diagnosis of ASH with concurrent atrophic UDT was considered.

**Figure 4:**
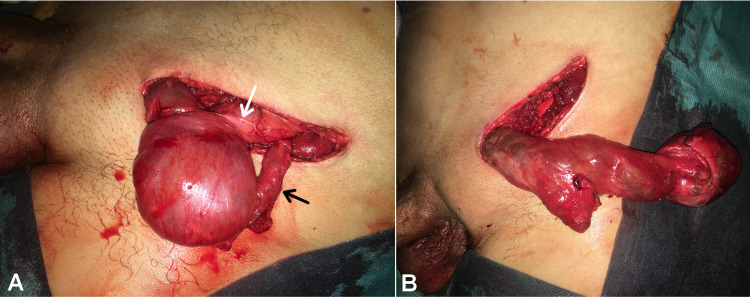
Intraoperative view image showing radical en-bloc excision of the ASH via an extended inguinal approach. (A) Proximal dissection of the retroperitoneal sac from its abdominal attachments; the peritoneum (white arrow) and the spermatic cord (black arrow). (B) Distal complete mobilization of the scrotal sac.

**Figure 5:**
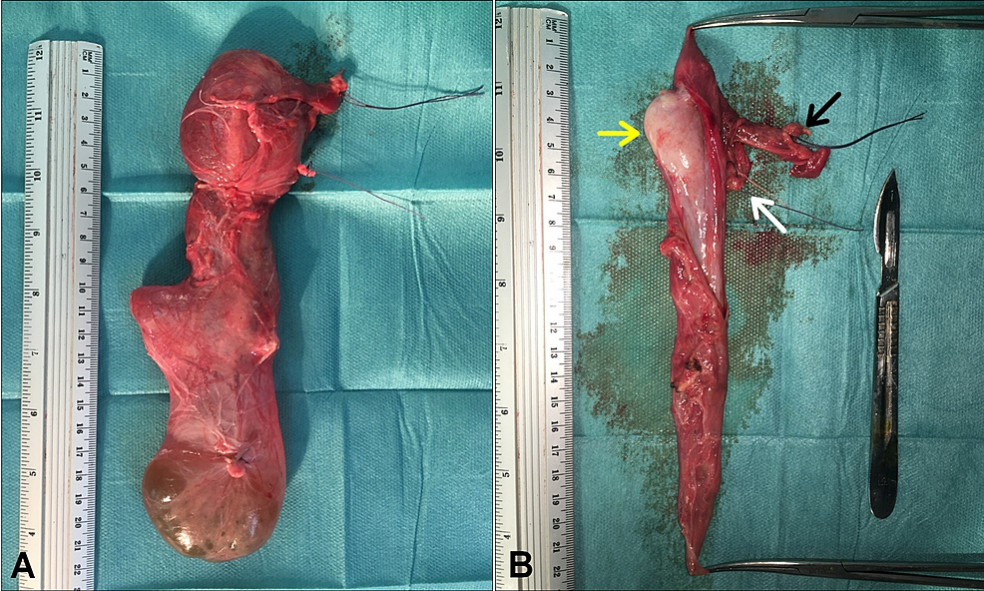
Macroscopic image showing gross specimen of the ASH. (A) Intact dumbbell-shaped cyst (B) Cut section showing an intracystic testis (yellow arrow) and its structures; the spermatic cord (black arrow) and the vas (white arrow).

The patient consented to undergo en-bloc excision of the ASH and UDT. He underwent an open inguinal incision, which was extended to the lower abdominal quadrant to reveal a patent spermatic cord opening into a retroperitoneal sac (Figure 4). No associated hernia sac was identified. The intact specimen was dissected extraperitoneally and resected completely (Figure 5), and the deep inguinal ring closed. The ASH contained an atrophic oval-shaped testis and 255 ml of clear-yellow free serous fluid with no septum between the two sacs. The post-operative period was uneventful.

Final histopathology of the testis and its tunica vaginalis, as well as cytology of the fluid, did not show any evidence of malignant transformation. At his two-year follow-up, the patient had no evidence of recurrence, hernia, or malignancy.

## Discussion

The incidence of coexisting UDT with ASH seems to be underestimated but shows a marked pediatric predominance. In this setting, the most common location is inguinal but rarely intra-abdominal [6,7]. The current case describes an aged man with late development of ASH, whereupon a neglected intra-abdominal UDT was found within the abdominal sac. Indeed, cryptorchidism is a congenital anomaly that may go unnoticed or have its management denied by parents who are unaware about potential risks of subfertility and malignant degeneration in adulthood, especially in developing countries”. However, ASH is an uncommon presentation of neglected UDT, which remains questionable as an etiological factor. 

Our observation of late development of a scrotal component in our patient, who initially had only inguinal swelling for three months, supports Sasidharan et al. hypothesis [8]. Accordingly, we assumed that the hydrocele had initially developed within the abdominal cavity as a hydrocele of intra-abdominal UDT. Moreover, the finding of an intra-abdominal sac, containing the UDT and lying posteriorly to the peritoneal sac, corroborates this presumption. We also assumed that under increased intra-abdominal pressure, the empty deep inguinal ring had acted as a lead point for downward displacement and herniation of an over distended abdominal hydrocele along the inguinal canal to form an inguinal swelling. After three months, the abdomino-inguinal hydrocele had prolapsed into a compliant empty left hemiscrotum, which had allowed the scrotal sac to be unusually larger than the abdominal one. Had this hydrocele shown only inguinal extension of a previously preformed intra-abdominal sac, it would have been named “hydrocele en bi-sac inverse” [5].

Most cases of adult ASH exhibit an upward properitoneal expansion of a preformed scrotal hydrocele [1-3,9], which may cause visible and palpable abdominal swelling. Thus, ASH can often be diagnosed through bimanual palpation of a hydrocele and abdominal mass that are cross-fluctuant. The “Springing back ball sign” is more characteristic than the cross fluctuation test, in which compression of the scrotum makes the abdominal component expand, but once pressure is released, the fluid spring back into the scrotum that regains its initial size [1-3]. In cases of small properitoneal sacs and in pediatric ASHs that usually harbor retroperitoneal expansion, ASH may go unnoticed until being incidentally discovered during ultrasound assessment or laparoscopic repair of presumptive inguino-scrotal hydrocele, respectively [4,10]. In this case, the abdominal sac had remained clinically silent until its distal expansion from the retroperitoneum into a previously empty left hemiscrotum. Moreover, it was neither visible nor palpable, so that the cross fluctuation and springing back ball manoeuvers were inconclusive, and imaging has played an important role in the incidental finding of an ASH.

The intercommunication between the two cystic compartments can be confirmed radiologically by ultrasound evaluation [1-5]. However, if USG is inconclusive or when complications are suspected, CT and MRI may be useful to better assess the abdominal cyst and its effect on surrounding tissues, as well as testis structural features [2,7]. They may also be helpful in unclear cases or disrupted anatomy, as in this case with unexpected clinical suspect of ASH in the setting of neglected ipsilateral cryptorchidism.

Moreover, our patient who presented with an intra-scrotal displacement of a clinically unnoticed retroperitoneal hydrocele could have been misdiagnosed as indirect inguinal hernia, which is the most common differential diagnosis in such clinical scenario [4-5,7]. Furthermore, in a patient presenting with inguinoscrotal swelling and neglected cryptorchidism, a germ cell tumor must also be considered. Other differential diagnoses include spermatic cord lymphangioma, abdominoscrotal spermatocele, giant hydronephrosis extending into the true pelvis, bladder diverticulum, and pelvic neuroblastoma [5,7].

If untreated, beyond any cosmetic issues, ASH may lead to complications that are mainly related to its long-standing compressive effect. It includes hydronephrosis, testicular dysmorphism, impaired spermatogenesis, deep vein thrombosis, leg edema, infection, testicular torsion, heamatocele, rupture, and risk of malignant transformation although anecdotal [1-3,11]. Our patient was told that his testicular atrophy most likely resulted from an underlying congenital abnormal development of the affected testis and its subsequent long-standing cryptorchidism rather than to be caused by ASH itself.

There are currently no guidelines on the management of ASH. Nevertheless, treatment is surgical as a spontaneous resolution is rarely reported in adults. The scrotal component is traditionally managed by plication, partial excision, eversion, or marsupialization of the tunica vaginalis. However, combined complete excision of the abdominal sac is mandatory to prevent recurrence and malignant transformation [1-3,11]. Moreover, removal of the normally positioned testis may be required in selected adult cases (i.e., challenging dissection of the spermatic cord hardly attached to ASH with a potential risk of testis necrosis) [12]. In the current patient, total excision of ASH may be carried out alone without orchiectomy, as it seemed feasible, safe, and effective. Nevertheless, the benefits of radical en-bloc excision of the ASH and UDT far outweighed the risk of developing germ cell tumors years later, especially in adult patients with long-standing post-pubertal cryptorchidism. Furthermore, it is important to distinguish “truly” UDT from an acquired up migration of a normally descended testis [13], as the latter may result from displacement into the abdominal sac following spontaneous rupture of the ASH, and maybe managed conservatively by orchidopexy.

In this case, an extended inguinal incision was carried out, which is the most used approach in ASH repair [1-5]. It helped us in improving the exposure of the retroperitoneal sac, identifying the spermatic cord and vas, making safe ASH mobilization from its peritoneal attachments, and keeping the whole sac intact as long as possible to facilitate its dissection. Others reported surgical approaches can be broadly categorized in inguinal, combined laparoscopic with inguinal or scrotal, pure laparoscopic, scrotal, and transperitoneal [2,4-5].

## Conclusions

To our knowledge, neglected intra-abdominal UDT presenting as ASH in the elderly has never been described in the literature. The most acceptable mechanism, although etiologically controversial and uncommon in adulthood, is an intra-scrotal expansion of the preformed retroperitoneal hydrocele of a UDT. As this retroperitoneal component may be neither visible nor palpable, ASH could be misdiagnosed as an indirect inguinal hernia. Thus, imaging assessment of the lower abdomen is crucial in suspected or unclear cases. While testis preservation can be used in selected ASH cases (i.e., pediatrics), radical en-bloc excision of the ASH and the testis is an acceptable alternative treatment in this condition. This case also favors the notion that an open surgical approach via extended inguinal incision may be superior to a pure inguinal one, providing efficient, secure, and effective procedure when approaching aged patients with concurrent neglected cryptorchidism.
